# Animal Safety, Toxicology, and Pharmacokinetic Studies According to the ICH S9 Guideline for a Novel Fusion Protein tTF-NGR Targeting Procoagulatory Activity into Tumor Vasculature: Are Results Predictive for Humans?

**DOI:** 10.3390/cancers12123536

**Published:** 2020-11-26

**Authors:** Wolfgang E. Berdel, Saliha Harrach, Caroline Brand, Kathrin Brömmel, Andrew F. Berdel, Heike Hintelmann, Christoph Schliemann, Christian Schwöppe

**Affiliations:** Department of Medicine A Hematology and Oncology, University Hospital Muenster, D-48149 Muenster, Germany; s.harrach@gmx.net (S.H.); Caroline.Brand@ukmuenster.de (C.B.); kathrin.broemmel@ukmuenster.de (K.B.); andrew.berdel@ukmuenster.de (A.F.B.); Heike.Hintelmann@ukmuenster.de (H.H.); Christoph.Schliemann@ukmuenster.de (C.S.); christian.schwoeppe@uni-muenster.de (C.S.)

**Keywords:** non-clinical safety and toxicology studies, vascular targeting, fusion protein tTF-NGR, regulatory requirements for translation of anti-cancer drugs into human trials, seamless phase 0 micro-dosing and phase I trial concept

## Abstract

**Simple Summary:**

Non-clinical safety, toxicology, and pharmacokinetic studies according to ICH guidelines with a new fusion protein tTF-NGR consisting of human truncated tissue factor (TF) and a small targeting peptide are reported. Results are compared with those of a phase I clinical dose escalation trial with tTF-NGR in cancer patients. Most of the non-clinical results were not predictive for human tolerability. Thus, animal sparing alternative pathways for translation of such a bio-pharmaceutical compound from preclinical studies on efficacy and mode of action into the clinic are discussed.

**Abstract:**

Background: CD-13 targeted tissue factor tTF-NGR is a fusion protein selectively inducing occlusion of tumor vasculature with resulting tumor infarction. Mechanistic and pharmacodynamic studies have shown broad anti-tumor therapeutic effects in xenograft models. Methods: After successful Good Manufacturing Practice (GMP) production and before translation into clinical phase I, ICH S9 (S6) guideline-conforming animal safety, toxicology, and pharmacokinetic (PK) studies were requested by the federal drug authority in accordance with European and US regulations. Results: These studies were performed in mice, rats, guinea pigs, and beagle dogs. Results of the recently completed clinical phase I trial in end-stage cancer patients showed only limited predictive value of these non-clinical studies for patient tolerability and safety in phase I. Conclusions: Although this experience cannot be generalized, alternative pathways with seamless clinical phase 0 microdosing—phase I dose escalation studies are endorsed for anticancer drug development and translation into the clinic.

## 1. Introduction

We have developed a new class of bifunctional proteins which anchor an essential procoagulatory molecule—tissue factor (TF)—into tumor vessels via C-terminal binding-motif peptides to induce selective tumor vascular occlusion and infarction [1,2,3,4,5,6,7,8,9,10,11,12,13,14,15,16,17,18,19,20,21]. To this end the non-specific membrane anchor of TF was replaced by peptides such as the CD13-binding NGR motif GNGRAHA. CD13 is an aminopeptidase selectively expressed on stimulated and growing endothelial cells (EC), such as on tumor EC with only limited presence on normal mature vascular tissue [22]. In some normal tissues such as small bile ducts, expression of CD13 furthermore does not necessarily impede application of CD13-targeted truncated TF (tTF), since this molecule is active only in a coagulation-competent environment such as in blood vessels. Combined anatomical and functional targeting provides the basis for cancer selectivity of these fusion proteins.

Preclinical mechanistic and pharmacodynamic studies in vitro and in vivo in particular with the lead fusion protein tTF-NGR [1,2,3,4,5,6,7,8,9,10,11,12,13,14,15,16,17,18,19,20,21] had shown:

- procoagulatory activity of tTF-NGR similar to TF in a factor X/factor Xa assay,
- specific binding to the respective target molecules (CD13) on stimulated EC,
- in vivo intratumoral accumulation shown with imaging techniques,
- in vivo induced intratumoral activation of coagulation with consecutive tumor vascular occlusion and inhibition of tumor vessel blood flow,
- in vivo therapeutic antitumor activity in xenotransplants and mouse tumor models independent of tumor histology,
- combinatorial activity with cytotoxic drugs and radiotherapy when applied in a specific sequence.

After setting up a Good Manufacturing Practice (GMP) process and obtaining a manufacturer’s authorization, translation into clinical phase I was planned and discussed with federal drug authorities (Paul Ehrlich Institute, Langen, Germany, PEI). Although we had performed acute and chronic toxicology studies in mice and already had clinical experience with micro-dosing of a few single end-stage cancer patients [5] according to the Declaration of Helsinki [23], scientific advice from the federal drug authority on the basis of and in agreement with the European regulations requested to perform additional animal safety and toxicology studies according to ICH M3 [24] and S6 (as modified in S9; [25]) guidelines of the European Medicines Agencies (EMA) before starting a phase I trial in cancer patients.

Following completion of these studies and a clinical phase I trial with tTF-NGR in late stage cancer patients beyond standard treatment [26], we here briefly summarize the results of these additional non-clinical studies and ask the question whether they have contributed significantly to the safety of first-in-human clinical application, or whether it was a long and expensive effort condoning the wasting of animal lives without essential impact on patient safety.

## 2. Materials and Methods

### 2.1. Academic Toxicology Studies for Intravenous Application of tTF-NGR in Mice

In parallel to the mechanistic and pharmacodynamic studies characterizing the therapeutic potential and the mode of action of tTF-NGR, safety studies and toxicology studies to monitor local reactions at the injection site and the body weight of the animals during treatment, to assay pathology and histology of different organs after treatment, and to establish the lethal dose for 10% of the treated animals (LD_10_) within 24 h were performed. Either male or female CD-1 or BALB/C mice for the LD_10_ study, or tumor xenograft-bearing nude (athymic) CD-1 or BALB/C mice for the therapy trials were used. As the nude BALB/C mice initially used were rather sensitive to any experimental procedure and also to targeted tissue factor fusion proteins, most experiments were done with athymic CD-1 mice. The C57BL6 mice strain was chosen as syngeneic mouse model. Mice were kept in our central animal facility under standard conditions as described [1,2,3,4,5,6,7,8,9,10,11,12,13,14,15,16,17,18,19,20,21]. Mice studies were performed in agreement with German regulations (Tierschutzgesetz § 8) and specifically approved in the form of a project license. Further methodological details of these studies have been described [5,15].

### 2.2. Safety Pharmacology and Toxicology Studies According to ICH S9 and S6 Guidelines

Although ICH M3 guidelines contain chapters elaborating on the possibility of exploratory clinical trials ([24], chapter 7), the scientific advice by PEI was interpreted by us in a conservative way. Thus, in addition to the academic mouse toxicology studies, mandatory formal extramural safety pharmacology and toxicology studies according to S9 [25] guidelines of the European Medicines Agencies (EMA) were performed at an external, professionally certified GLP (Good Laboratory Practice) contract laboratory. All studies on safety pharmacology, and toxicology were performed under specific institutional GLP licenses by the responsible German State Authority for Health and Consumer Protection. Results from the official reports are published with approval by this laboratory. The compound tTF-NGR was tested in 4 species. CD-1 mice, CD-1 rats, and Dunkin-Hartley guinea pigs were obtained from Charles River Laboratories (69592 L’Arbresle Cedex, France or 97633 Sulzfeld, Germany). Animals were kept under standard conditions with water and food ad libitum.

CD-1 mouse study 1 (Table 1) was an examination of the pulmonary parameters (breaths/min, peak inspiratory flow, peak expiratory flow, inspiratory time, expiratory time, and airway resistance index) for a period of 4 h after application of tTF-NGR using whole body plethysmography and clinical examination upon 1-h i.v. infusion of 0, 1.8, 6.0, and 18.0 mg tTF-NGR/m^2^ body surface area (b.s.a.); *n* = 40). The study was performed by using a parallel positive control group with subcutaneous application of 30 mg carbamyl-beta-methylcholine chloride (betanechol). Study 2 was a neuropharmacological screening study in CD-1 mice (*n* = 32) according to IRWIN with 40 standard neurological parameters (behavioral reactions, motor activity, CNS, posture, motor coordination, muscle tone, and reflexes) measured following 1-h i.v. infusion of doses identical to study 1 and over a period of 2 h after end of application. Study 3 was a CD-1 mouse dose escalation tolerability study performed by intravenously (i.v.) injecting single doses of 0, 6.0, 20.0, and 60.0 mg tTF-NGR/m^2^ b.s.a. (*n* = 12) using standard clinical parameters.

Study 4 in rats was a dose escalation tolerability study using 1-h i.v. infusions of single doses of 12, 36, 60, and 120 mg tTF-NGR/m^2^ b.s.a. (*n* = 10) using standard clinical parameters.

Study 8 in guinea pigs was examining skin sensitization according to Magnusson and Kligman (maximization test; according to EC method B.6. (Regulation (EC) No. 440/2008) and OECD guideline 406) in guinea pigs (*n* = 30) repeatedly applying undiluted stock solution of tTF-NGR (681 microgram tTF-NGR/mL) intracutaneously. Study 9 was testing guinea pigs (*n* = 5) for non-antigenicity on day 46 of the experiment in animals previously sensitized 6 times intraperitoneally with stock solution of tTF-NGR according to U.S. Pharmacopeia.

Although the ICH S9 guideline applicable for non-clinical studies designed to prepare clinical trials in cancer patients is more flexible than the requirements of ICH M3 and S6, it still carries non-rodent in addition to rodent studies. For enhanced predictability for human safety, beagle dogs were chosen as the non-rodent species, as they are a widely accepted model when studying human tissue factor and coagulation processes in animals [27,28,29,30,31,32,33,34,35]. Beagle dogs were obtained from Marshall BioResources (North Rose, NY 14516, USA) and kept under standard conditions with water and food at libitum. Study 6 (*n* = 6) was testing the cardiohemodynamic effects of tTF-NGR in telemetered beagle dogs following single i.v. 1-h infusions of 0.0, 2.0, 6.0, and 20 mg/m^2^ b.s.a. of the drug. Among the parameters evaluated besides clinical behavior were ECG parameters such as heart rate, RR interval, QRS complex and interval, QT interval, QTc values (van de Water, Fridericia), the PQ interval, and any form of arrhythmia. Study 5 (*n* = 6) was an orienting Maximum Tolerated Dose (MTD) study of tTF-NGR using single i.v. infusions and following daily 1-h i.v. infusions for 5 days of 20.0, 40.0, and 80.0 mg/m^2^ b.s.a. (2 dogs per cohort), accompanied by continuous clinical and repeated blood count, blood chemistry, and toxicokinetic analysis. Finally, study 7 was a 5-day subchronic toxicity study of tTF-NGR testing daily 1-h i.v. infusions for 5 days of 0.0, 2.0, 6.0, and 20.0 mg/m^2^ b.s.a. (*n* = 32; male and female animals per dose cohort) and measuring a broad panel of clinical, cardiovascular (including ECG, RR), ophthalmological, and auditory examinations, hematology, hemostaseology, clinical biochemistry, urinalysis, postmortem pathology and histopathology, and pharmacokinetic examinations.

### 2.3. Pharmacokinetic and Toxicokinetic Studies

Pharmacokinetic and toxicokinetic studies of tTF-NGR in beagle dog plasma were performed in studies 5 and 7 according to a modified ELISA-method validated according to GLP guidelines with regard to accuracy, precision (repeatability and intermediate precision), long-term and freeze-thaw stability in the external laboratory. The methods including evaluation tools and software were essentially identical to the ones validated by the authors and then used in the clinical phase I trial (PKsolver; [26]). The studies on toxicokinetics were also performed under specific institutional GLP licenses by the responsible German State Authority for Health and Consumer Protection.

### 2.4. Clinical Study

The clinical phase I study with tTF-NGR was recently published in detail [26]. The protocol (with amendments) was approved by the Ethical Board of the Physicians’ Chamber of Westphalia-Lippe and the Westphalian Wilhelms University of Muenster (AZ 2016-414-f-A) and by the PEI. Written informed consent by the patients was obligatory prior to entry to the study.

## 3. Results

### 3.1. Academic Toxicology Studies for Intravenous Application of tTF-NGR in Mice

The high selectivity of vascular occlusion by tTF-NGR for tumor blood vessels was demonstrated by the fact that no visible thrombosis occurred in the vasculature of normal tissues such as heart, kidney, liver, and lung at therapeutic doses [1,5]. In general, toxicity of tTF-NGR at therapeutic dose levels was low. There were occasional deaths in all groups of nude mice during experiments occurring without clear dose–relation within the dose range used for therapy. Safety evaluations with pathohistology were repeatedly done in all therapeutic experiments and revealed a systemic No-Observed-Adverse-Effect-Level (NOAEL) for tTF-NGR of >1 mg/kg × 6 (equivalent to >3 mg/m^2^ (×6) body surface area (b.s.a.)).

**Figure 1:**
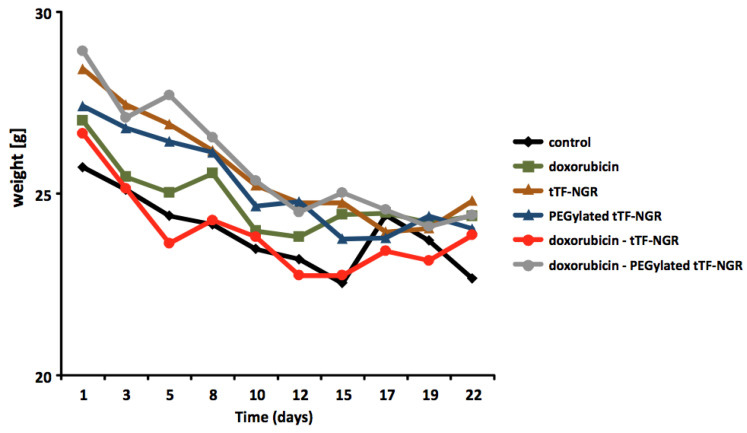
Body weight curves of athymic mice during combination experiments with tTF-NGR. Body weight curves of athymic mice during combination experiments with tTF-NGR (1 mg/kg b.w. × 2), PEGylated tTF-NGR (5 mg/kg b.w. × 2), and the combination of these compounds with doxorubicin (5 mg/kg b.w.). One representative experiment of multiple from Reference 15 is depicted.

Body weight measurements of the tumor-bearing athymic mice were repeatedly performed during the therapeutic experiments. Figure 1 depicts one of multiple such body weight curves measured during the therapy experiments and is taken from experiments published [15] comparing i.v. saline control, tTF-NGR, PEGylated tTF-NGR, doxorubicin and combinations of doxorubicin with either tTF-NGR or the PEGylated tTF-NGR. While slight reductions of body weight during the experimental procedure were often observed, there were no differences between saline (PBS) controls and the single experimental groups. Thus, the weight loss was attributed to the general tumor stress, but not to the specific test compounds used. We observed tail tip necrosis after repeated intravenous (i.v.) application occurring at 1 mg/kg body weight (b.w.) (i.v.). This and the volume limitation for i.v. application to mice prevented us from increasing the dose beyond 1.5 mg/kg b.w. (i.v.) for the repeated dosing in the therapeutic experiments.

**Figure 2:**
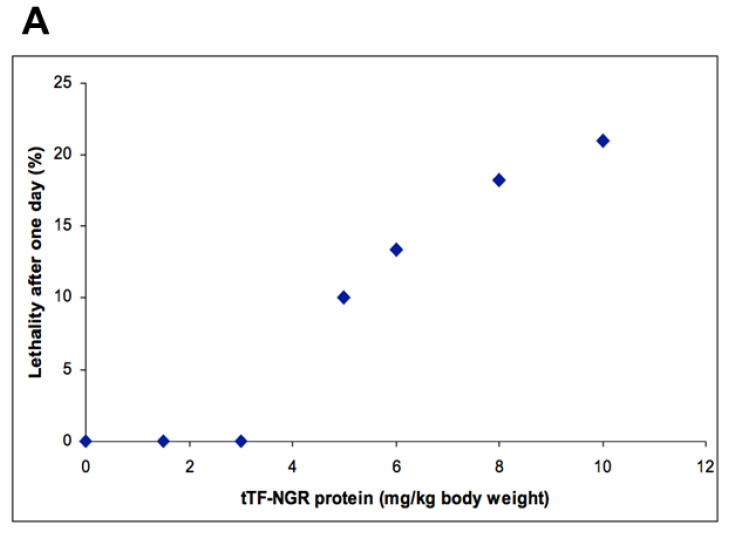

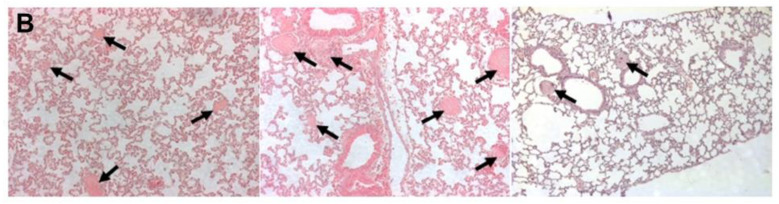
Effects of tTF-NGR on normal (non-athymic) CD-1 or BALB/C mice. (**A**) Lethality given as percentage of mice treated (1 day observation period) for normal (non-athymic) CD-1 or BALB/C mice (2–5 months old) upon i.v. injection of tTF-NGR at the doses of 0.0, 1.5, 3.0, 5.0, 6.0, 8.0, and 10.0 mg tTF-NGR/kg body weight. LD10 (lethal dose for 10 % of the animals treated) was 5.0 mg/kg or 15.0 mg tTF-NGR/m^2^ b.s.a. (**B**) H & E staining of lungs of mice that died upon treatment with toxic doses of tTF-NGR. Blood vessels are thrombosed (arrows).

Dose-escalation studies in normal (non-athymic) CD-1 or BALB-C mice with single i.v. injections revealed i.v. LD_10_ (lethal dose for 10% of the animals) as being ≥5 mg/kg (equals ≥15 mg/m^2^ b.s.a.; Figure 2A). At these doses some of the dead animals showed pulmonary embolism in postmortem histology (Figure 2B) and non-specific histological signs of cerebral toxicity. All other organs remained without visible toxicities.

In these dose-escalation studies in mice, a formal NOAEL including pathohistology was not established, as only the animals who died from the treatment at the different dose levels were studied in detail including pathology and pathohistology. The others were clinically observed for approx. 4 weeks. In these animals, the clinical NOAEL was 3 mg/kg b.w. × 1 (equivalent to 9 mg/m^2^ b.s.a. × 1).

As stated above, a NOAEL in mice can also be defined from the previous therapeutic studies in the xenograft models with the A 549 lung cancer, the M21 melanoma, and the HT1080 fibrosarcoma, since these mice were regularly sacrificed at the end of the experiments and studied including histopathology. In these experiments there were no systemic signs of toxic effects. The NOAEL for tTF-NGR given i.v. repeatedly for up to 6 times every day or every second day in tumor-carrying nude mice was above 1 mg/kg (equivalent to 3 mg/m^2^ b.s.a. × 6). Information from academic preclinical in vivo studies was limited by the lack of further dose escalation.

### 3.2. External Safety Pharmacology and Toxicology Studies According to ICH S9 and S6 Guidelines

#### 3.2.1. Mouse Studies

In addition to the academic studies in mice, formal mouse studies were done by a GLP-certified professional contract laboratory. A dose-range-finding study by single i.v. infusion into CD-1 mice reproduced the local tissue side effects when tTF-NGR was injected into a small peripheral vein at high local concentrations. Systemic tolerance testing at 6, 20, and 60 mg/m^2^ b.s.a. with 4 animals at the 6 and 20 mg/m^2^ dose levels and with 2 animals at the 60 mg/m^2^ dose level revealed 1/4 deaths at the 20 mg/m^2^ and 2/2 deaths at the 60 mg/m^2^ dose level. The LD_10_ findings seen in the academic studies were reproduced in a larger cohort of mice. Clinical signs of tolerability problems occurred at every dose level and were reduced with lower application volume.

Examination of pulmonary function parameters employing whole body plethysmography following i.v. infusion of tTF-NGR revealed a NOAEL at above 18 mg/m^2^ b.s.a., which was the highest tested dose level of three (1.8, 6.0, 18.0 mg/m^2^) in this test.

Neuropharmacological screening according to IRWIN upon single infusions of tTF-NGR revealed a NOAEL at above 18 mg/m^2^ b.s.a., which was the highest tested dose level of three (1.8, 6.0, 18.0 mg/m^2^, 8 mice per group) in this test.

#### 3.2.2. Rat Studies

A dose-range-finding study of single dose tTF-NGR given as 1-h i.v. infusion was performed in CD-1 rats. The NOAEL was higher than 120 mg/m^2^ b.s.a. at which dose level the study was ended. The possible reason for the low toxicity in this species can hypothetically be deduced from amino acid sequence comparisons of the TF molecule among different species including humans in the essential KK region, which is functionally important for procoagulatory activity [36,37,38]. Contrary to humans, the rat has an RK sequence in this position [39], indicating that the factor VIIa binding and factor X activation might be suboptimal when using human material with a KK-region in this species.

#### 3.2.3. Guinea Pig Studies

Examination of tTF-NGR in the skin sensitization test according to MAGNUSON and KLIGMAN (maximization test) was performed with undiluted material. tTF-NGR was found to be non-sensitizing to guinea pigs.

Examination of tTF-NGR for non-antigenicity was performed in sensitized guinea pigs with undiluted material. Under the test conditions applied, tTF-NGR resulted in anaphylaxis in guinea pigs. This reflects the expected anaphylactic reaction to repeated applications of a recombinant (*E. coli*) xenogeneic protein with low amino acid sequence homology. The sequence homology of the tissue factor protein between humans and guinea pigs is 67%.

#### 3.2.4. Beagle Dog Studies 

A single infusion and a following 5-day Maximum Tolerated Dose (MTD) study (study 5, Table 1) was performed applying first single, then repeated 1-h i.v. infusions of tTF-NGR to beagle dogs. None of the animals treated with escalating dose levels of 20, 40, and 80 mg tTF-NGR/m^2^ b.s.a. by 1-h intravenous infusion for 1 day each, none of the treatment-naïve dogs treated with a single dose of 20 or 40 mg tTF-NGR/m^2^ by 1-h intravenous infusion, and none of the dogs treated by repeated dosing for 5 consecutive days with 20 mg tTF-NGR/m^2^ by 1-h intravenous infusion showed any signs of local intolerance reactions at the infusion. Systemic adverse test item-related effects were noted:

- upon one single application starting at 4 mg tTF-NGR/kg b.w., equivalent to 80 mg/m^2^ b.s.a. (changes in behavior).
- upon daily applications for 5 consecutive days starting at 1 mg tTF-NGR/kg b.w., equivalent to 20 mg/m^2^ b.s.a. (bile acids and bilirubin serum levels increased in both of 2 animals, ALAT serum activity increased and behavioral changes were observed in 1 out of 2 animals).
  No immunological effects consistent with decreasing or ameliorating the tolerance of secondary or follow-up applications were noted.

Under the test conditions of this study, the MTD was considered to be 1 mg tTF-NGR/kg b.w. (equivalent to 20 mg/m^2^ b.s.a.) by 1-h intravenous infusion when given daily for 5 days.

Cardiohemodynamic studies of the effects of tTF-NGR in telemetered beagle dogs following i.v. administration of tTF-NGR concluded that a single i.v. dose of 2, 6, and 20 mg/m^2^ b.s.a. did not have any effect on cardiovascular parameters in this species.

With the result of these orientation studies, the “toxic high” dose for the regular “subchronic toxicity” study was selected as being 20 mg/m^2^ tTF-NGR infused over 1-h daily for 5 days (Table 1, study 7). The animals were closely monitored including a broad laboratory program. In this 5-day subchronic toxicity study of tTF-NGR by repeated i.v. administration (1-h infusion) to beagle dogs no local or systemic toxic effects were observed. The macroscopic and histopathological inspection at necropsy did not reveal any test item-related morphological local or systemic organ changes. No test item-related influence was noted on the relative or absolute organ weights of the animals. Thus, the NOAEL for tTF-NGR in the dogs was stated as being above 20 mg/m^2^ b.s.a. for a daily × 5 infusion. Table 1 summarizes the non-clinical safety studies performed. Table 2 summarizes the key toxicology findings of these studies.

Since our quality testing of the GMP process for tTF-NGR ensures extremely low to absent concentrations of any excipients or impurities, the toxicities observed in these studies have to be interpreted as tTF-NGR related toxicities.

### 3.3. Pharmacokinetic (PK) and Toxicokinetic Studies in Beagle Dogs

Within beagle dog studies, but in particular within the 5-day subchronic toxicity study of tTF-NGR by repeated i.v. administration (1-h infusion) to beagle dogs (see Table 1, Studies 5 and 7), repeat toxicokinetic studies have been performed. The data are summarized as follows:

The C_max_-levels (maximum plasma concentration) and AUC (area under the curve) revealed a dose-related exposure of the animals to tTF-NGR during the treatment period. Mean peak plasma levels of tTF-NGR in the male and female animals treated with 0.1, 0.3 or 1.0 mg tTF-NGR/kg b.w./day by a 1-h intravenous infusion for 5 days (equivalent to 2.0, 6.0 or 20.0 mg/m^2^ b.s.a.) were generally observed 1-h after start of dosing with peak concentrations of 584, 1632, and 4777 ng/mL for the males, respectively, and 405, 1487, and 3468 ng/mL for the females, respectively. The calculated mean alpha plasma distribution half-life (t_1/2_) of tTF-NGR ranged from 0.84 to 1.51 h in males and from 0.78 to 1.79 h in females and the calculated mean terminal plasma elimination half-life of tTF-NGR ranged from 21.71 to 42.63 h in males and from 18.27 to 78.33 h in females. The individual terminal elimination plasma half-lives ranged from 13.79 to 50.80 h for the males and from 18.27 to 108.75 h for the females. All half-lives of the individual animals were similar except for the low dose animals, in particular one animal with 108.75 h. The slightly longer elimination half-life calculated for tTF-NGR for one animal of the low dose group (females; t_1/2_ 108.75 h) was due to a slightly uncertain evaluation of the half-life due to the fact that most of the terminal plasma levels were very close to the detection limit. Hence, no gender difference was noted for the elimination half-life. The dose proportion factor (DPF) of the AUC0-∞ value for the high dosed animals was 0.97 for the males and 1.07 for the females, and therefore, proportional. The individual and mean results of the toxicokinetic analysis of the test item tTF-NGR in dog plasma are given in Table 3.

**Figure 3:**
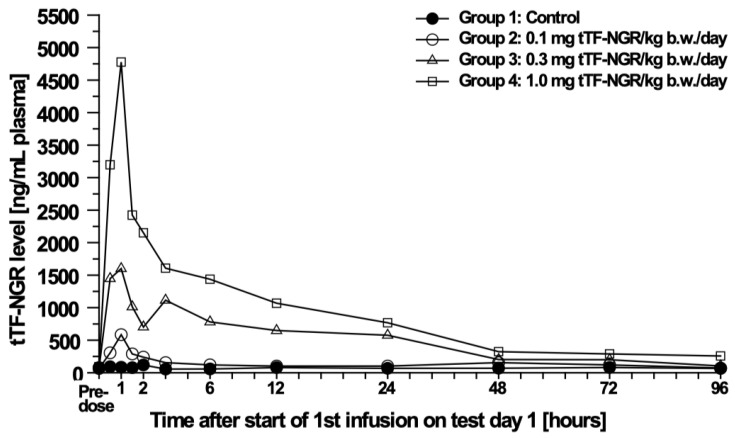
Mean plasma levels in male beagle dogs. Plasma levels in ng/mL after intravenous 1-h infusion of tTF-NGR (*n* = 3) at different doses (2.0, 6.0, and 20.0 mg/m^2^ b.s.a.).

Figure 3 shows the mean plasma levels in male beagle dogs after 1-h i.v. infusion of tTF-NGR as an example.

## 4. Discussion

### 4.1. Quality of the Non-Clinical Studies

The first and last authors are responsible for the academic studies in mice. They visited the external GLP contract laboratory twice to oversee the quality of work and the compliance with guidelines without any complaints and regularly discussed and co-planned every single study. On the other hand, interpreting and reporting the extramural study results was done by the scientists in the external laboratory without input of the authors.

### 4.2. Predictability of the Non-Clinical Studies for the Clinical Phase I Study in Cancer Patients

All non-clinical safety and toxicology studies were consulted to choose 1 mg/m^2^ b.s.a., given as daily 1-h i.v. infusions in 0.9% NaCl via a central venous line for 5 consecutive days with 2-week rest periods before the start of the next cycle as a presumably safe starting dose for clinical phase I. For justifying this safe starting dose, the safety data of the most sensitive non-human species, the mouse (see Table 2), and the data from the single patients treated before the formal non-clinical safety studies were most important [39].

The clinical phase I study allowed for intraindividual dose escalations between cycles and 17 patients were treated with at least one complete cycle of daily infusions over 5 days [26]. MTD defined as dose-level with Dose Limiting Toxicity (DLT) in <2 out of 6 patients treated in this phase I study was 3 mg/m^2^ tTF-NGR/day × 5, q day 22. DLT was an isolated and reversible elevation of high sensitivity (hs) Troponin T hs without clinical sequelae. Three thromboembolic events (grade 2), observed at the 3 mg/m^2^, 4 mg/m^2^, and 5 mg/m^2^ dose levels, respectively, were interpreted as tTF-NGR-related although other relevant risk factors were present, and were completely reversible upon anticoagulation. Otherwise, the tolerability was good and there were no treatment-related deaths on the trial. Details are published [26].

### 4.3. Predictive Value of Non-Clinical Safety and Toxicology Studies

In the following, we discuss which of the non-clinical data have helped to improve patient safety in this trial.

#### 4.3.1. Mouse Data

Amino acid sequence homology between human and mouse is approx. 60.09% and both species share the functionally important KK region [36,37,38,39], encouraging evidence transfer from mouse to human.

Local toxicity observed as tail tip necrosis in the injected athymic mice was important, since it prompted the obligatory application of tTF-NGR as a slow infusion via central venous line. After one catheter-associated subclavian thromboembolic event was observed in phase I and interpreted as possibly treatment-related, central-venous port catheters were made obligatory for application.

Systemic safety data in athymic mice were also predictive, since the NOAEL of 3 mg/m^2^ was close to human MTD. However, pulmonary embolism occurring at higher doses was dose-limiting in mice but not observed in humans. Among other reasons, this difference was possibly due to the extremely high C_max_ levels of tTF-NGR induced by the fast i.v. injection. C_max_ levels in the mouse were measured up to >10,000 ng/mL upon application of 3 mg/m^2^ [39], i.e., more than 12-fold of levels observed in the human plasma upon 1-h infusion of 3 mg/m^2^ tTF-NGR (788.74 ng/mL; [26]).

#### 4.3.2. Other Species

The non-clinical safety and toxicology data in the rat, the guinea pig, and also in the beagle dog were largely non-predictive for the human MTD and DLT. With NOAEL of >120 mg/m^2^ in the rat, tolerability in this species was not predictive for human safety, possibly due to rather low amino acid sequence homology of TF between rat and man (approx. 59.6%) and the difference in the region important for the procoagulatory activity (RK versus KK; 36–39). On the other hand, rat experiments indicated that dose-limiting toxicity is clearly linked to the procoagulatory function of the tTF-NGR molecule, since non-specific toxicity of the molecule with a minimal “non-fit” for procoagulatory efficacy in a chosen species yields very high doses of NOAEL.

The data in dogs, with No-Effect-Doses of >20 mg/m^2^ using daily injections, grossly underestimated toxicity seen in humans, although amino acid sequence homology of TF between human and dog is rather high (approx. 75.6%), both species share the functionally important KK region [39], and canine models are often recommended as adequate models to study procoagulatory effects of human TF [27,28,29,30,31,32,33,34,35]. Furthermore, in retrospective analysis of dog plasma from non-clinical experiments at all dose levels, elevation of high sensitivity (hs) Troponin T hs could not be found upon tTF-NGR application, and thus also qualitative DLT was not predicted. Of course, an elderly multimorbid cancer patient group bears a different cardio-vascular risk profile than a young healthy beagle group. On the contrary, retrospectively checking the individual patients’ charts of the 5 individuals within the micro-dosing therapy revealed transient grade 1 elevation of Troponin T hs in one patient who already had an elevated Troponin T hs before therapy, compatible with intra-species predictiveness.

#### 4.3.3. Pharmacokinetics (PK)

Mouse PK were largely non-predictive for the human situation due to the necessity of bolus i.v. injection of the protein instead of slow 1-h infusion (details in 39). Comparing dog with human PK, there were some similarities. C_max_ levels were similar at equal doses (2 mg/m^2^: 584.3 ng/mL for male and 405 mg/mL for female dogs versus 341.3 ng/mL in humans). However, as the mean terminal half-life differed (2 mg/m^2^: 42.63 h for male and 78.33 h for female dogs versus 7.4 h for humans) which contributed to approx. double AUC values for dogs compared to human, the dog PK predicted accumulation occurring with daily applications, whereas the human PK excluded accumulation. In addition, whereas DPF mirrored proportionality in dogs, the human DPF was >1.0 indicating an elimination lower than proportional with higher doses.

### 4.4. Central Statement and Consequences to Be Discussed

In parallel to the broad mechanistic and pharmacokinetic studies on the antitumor efficacy and mode of action of tTF-NGR as the lead structure of a new class of antitumor fusion proteins, studies in xenograft-tumor bearing athymic mice provided some predictive safety information for the human situation. With this exception, data obtained from formal ICH S9 and S6 animal safety, toxicology, and pharmacokinetic studies were not predictive for patient safety in the first-in-human clinical trial. This central summary statement refers only to the fusion protein tTF-NGR and can of course not be generalized.

The complexity of defining NOAEL and its meaning for clinical safety and reproducibility in clinical trials has been controversially discussed for a long time [40,41,42,43]. Nevertheless, although the ICH S9 guideline certainly has considerably down-sized previous guidelines for preparing cancer patient trials and provides a flexible framework, this guideline still generalizes requirements, and the necessity of this in our view has to be reconsidered. Indeed, the S9-guided experiments reported here were time-consuming (>1 year), expensive (approx. 600,000 Euros), misleading, and wasted animal lives without increasing patient safety in phase I.

Most importantly, before performing these non-clinical studies, we had treated 5 individual patients suffering from advanced cancer beyond any standard therapy and an estimated survival time of few weeks to months. After obtaining legal and ethical advice and with their informed consent these patients were treated with single micro-doses of tTF-NGR between 1 and 4 mg/m^2^ b.s.a. (5, 39). This was done in strict accordance with the Declaration of Helsinki [23] with the objective to offer a last therapeutic chance to these individuals in an end-stage situation. Since we did not reach this objective for these individuals, we then stopped single patient application in this low dose-range without DLT and concentrated on further systematic translational development of the compound. The data from these micro-dosing case histories later contributed to the finding of a safe starting dose for clinical phase I [39].

This manuscript has no intention to take a general position on ethics of animal experiments, nor on the strengths and pitfalls of certain model systems. The literature is full of examples discussing anthropocentric versus animal rights views [44,45] and societies are under enormous pressure to reprioritize their legal frameworks. In our view, animal experiments certainly form the basis for much of our medical progress in developing desperately needed diagnostics and therapeutics. This publication adds another compound to the many cytotoxic and biopharmaceutical compounds, for which—besides ethical concerns—there are also serious scientific limits to the predictive value of guideline-conforming animal toxicology data for humans (for further ref. see 40–45). Nevertheless, these guidelines still can be interpreted as being an obligatory part of regulatory frameworks in Europe and the US. Such non-clinical toxicology studies take a huge effort and may hinder cancer patients’ access to investigational new drugs. This in our view must be distinguished from the necessity to obtain in vivo mechanistic and pharmacodynamic data on therapeutic activity and mode of action of an investigational anti-cancer drug before planning translation from the laboratory to the clinic. With in vitro experiments only, oncology would have missed the “prodrug” cyclophosphamide to name only one example.

## 5. Conclusions and Alternative Options for a Translational Procedure in Oncology

As exemplified here with a new targeted fusion-protein, results of formal preclinical animal safety and toxicity studies according to EMA (and FDA) guidelines M3, S6 and S9 are not generally predictive for subsequent human trials and thus do not necessarily contribute to patient safety. On the contrary, they may impede anti-cancer drug development. Thus, with the exception of gene therapy and immune therapy, e.g., with species-specific antibodies involved, we endorse an alternative translational procedure for anti-cancer drugs: safety studies in parallel to the mechanistic and pharmacodynamic studies in one appropriate species should be obligatory. As the necessity for further toxicology studies probably varies between different drug candidates and also on the basis of these orienting safety studies, the necessity for additional non-clinical safety and toxicology should be discussed and planned within the scientific advice by investigators and federal regulatory drug authorities individually for each investigational compound with the objective to considerably reduce non-clinical toxicology testing. This approach is already discussed in chapter 7 of ICH M3 [24] and the “Exploratory IND studies” guideline of the FDA [46]. As proposed and reviewed by others [47], the first-in-human study in oncology according to the Declaration of Helsinki, the Good Clinical Practice (GCP) guidelines and within the European drug legislation could be a seamless approach between phase 0 micro-dosing of few patients for establishing a safe starting dose and a following phase I dose escalation. In such a seamless clinical phase 0—phase I study the safe starting dose for the dose escalation part might often be lower and more cautious as within the procedure practiced today. Then, to enable more patients to be treated in the upper and potentially active dose levels of the drug candidate, intra-individual dose escalation from cycle to cycle could be allowed within the first and lower dose levels. Doing this, fewer patients might be needed to approach MTD, and the study protocol could carry provisions for switching to a traditional Fibonacci 3 + 3 design at higher dose levels.

## Figures and Tables

**Table 1:** List of toxicology studies performed according to ICH S9 (and S6) guidelines during the development of tTF-NGR.

Type of Study/Dose Duration	Administration Route	Species (tTF-NGR Dosage)	Animals *n*/ Sex/Group
**Single Dose**			
In-house UKM	i.v.	mice (DRF, e.g., Figure 2)	101/F+M
Study 1	i.v.	mice (DRF 0; 1.8; 6.0; 18.0 mg/m^2^)	40/F
Study 2	i.v.	mice (DRF 0; 1.8; 6.0; 18.0 mg/m^2^)	32/F
Study 3	i.v.	mice (DRF 6; 20; 60 mg/m^2^)	12/F
Study 4	i.v.	rats (DRF 12; 36; 60; 120 mg/m^2^)	10/F
Study 5	i.v.	beagle dogs (DRF 20; 40; 80 mg/m^2^)	6/F+M
Study 6	i.v.	beagle dogs (DRF 0; 2; 6; 20 mg/m^2^)	4/F+M
**Repeat Dose**			
In-house UKM	i.v.	mice (1.0; 1.5 mg/kg b.w.)	in all therapeutic exp./F+M
Study 7	i.v.	beagle dogs (0; 2; 6; 20 mg/m^2^)	32/F+M
Study 8	i.c.	guinea pigs (stock solution)	30/M
Study 9	i.p., i.v.	guinea pigs (stock solution)	5/M
**Genotoxicity** NA			
**Reproductive Toxicity** NA			
**Local Tolerance**			
In-house UKM	i.v.	mice (1.0; 1.5 mg/kg b.w.)	in all therapeutic
			exp./F+M
Studies 5, 7	i.v.	beagle dogs (DRF)	see above
**Other Toxicity** Studies 8, 9: Non-antigenicity + sensitization		Guinea pigs (stock solution)	see above

Key: b.w. = body weight; DRF = dose range finding; i.c. = intracutaneous; i.p. = intraperitoneal; i.v. = intravenous; M = male; F = female; *n* = number of animals; NA = not applicable. For further details see Materials and Methods.

**Table 2:** Summary of key toxicology findings for tTF-NGR.

	Mouse		Rat		Dog	
Findings	Effect Dose (mg/m^2^)	No Effect Dose (mg/m^2^)	Effect Dose (mg/m^2^)	No Effect Dose (mg/m^2^)	Effect Dose (mg/m^2^)	No Effect Dose (mg/m^2^)
Xenograft therapy trials		>3 (x6)				
Academic	>/=15 * LD_10_	9 *				
toxicology						
	local at all doses;					
Study 3	systemic	systemic 6 *				
	20 *					
Study 1		>18 *				
Study 2		>18 *				
Study 4				>120 *		
Study 5					20 (x5)	
Study 7						>20 (x5)

* = single application.

**Table 3:** Non-compartment analysis of tTF-NGR in beagle dogs.

Non-Compartment Analysis of to tTF-NGR Test Day 1 (up to 24 h after Start of 1st Infusion)									
Dose [mg/m^2^]	Animal no.	C_max_^#1^ [ng/mL]	t _max_^#1^ [h]	t_1/2 alpha_ [h]	t_1/2 terminal_ [h]	K_el_ [1/h]	AUC_0-t last_ [ng*h/mL]	AUC_0-t last_/dose [h*kg*ng/mL/mg]	DPF
**Males**									
**2.0**	11	723.6	1.0	1.17	40.23	0.02	3712.05	37,120.50	
	12	531.2	1.0	1.12	36.87	0.02	2867.16	28,671.60	
	13	498.2	1.0	1.14	50.80	0.01	2922.71	29,227.10	
	**mean**	**584.33**	**1.00**	**1.14**	**42.63**	**0.02**	**3167.31**	**31,673.07**	n.a.
**6.0**	17	1613.9	1.0	0.88	20.85	0.03	19,250.05	64,166.83	
	18	1567.5	1.0	0.88	24.83	0.03	17,272.91	57,576.37	
	19	1715.8	0.5	0.77	37.11	0.02	16,311.34	54,371.13	
	**mean**	**1632.40**	**0.83**	**0.84**	**27.60**	**0.03**	**17,611.43**	**58,704.78**	1.85
**20.0**	23	5335.5	1.0	1.54	28.15	0.02	31,557.99	31,557.99	
	24	4533.8	1.0	1.05	23.20	0.03	29,021.97	29,021.97	
	25	4462.5	1.0	1.94	13.79	0.05	31,633.30	31,633.60	
	**mean**	**4777.27**	**1.00**	**1.51**	**21.71**	**0.03**	**30,737.85**	**30,737.85**	0.97
**Females**									
**2.0**	14	385.5	1.0	0.86	47.90	0.01	2137.61	21,376.10	
	15	330.7	1.0	0.80	-	-	2020.55	20,205.50	
	16	500.3	1.0	0.67	108.75	0.01	2022.04	20,220.40	
	**mean**	**405.05**	**1.00**	**0.78**	**78.33**	**0.01**	**2060.07**	**20,600.67**	n.a.
**6.0**	20	1624.9	1.0	1.68	18.27	0.04	15,059.23	50,197.43	
	21	1292.5	1.0	2.49	-	-	12,383.58	41,278.60	
	22	1542.8	1.0	1.20	-	-	14,870.10	49,567.00	
	**mean**	**1486.73**	**1.00**	**1.79**	**18.27**	**0.04**	**14,104.30**	**47,014.34**	2.28
**20.0**	28	3274.7	1.0	1.60	18.31	0.04	21,901.66	21,901.66	
	29	3819.7	1.0	1.51	18.40	0.04	21,570.02	21,570.02	
	30	3309.1	1.0	1.19	28.80	0.02	22,450.51	22,450.51	
	**mean**	**3467.83**	**1.00**	**1.43**	**21.84**	**0.03**	**21,974.06**	**21,974.06**	1.07

#1, values obtained from plasma analysis, all other values calculated by toxicokinetic analysis; n.a., not applicable; -, calculation of parameter not possible as no decrease of plasma levels was observed in time interval 3 to 24 h; AUC, area under the curve; C_max_, maximum concentration; DPF, dose proportion factor; K_el_, elimination rate constant; t_1/2_, plasma elimination half-lives (alpha (distribution phase), terminal (elimination phase) half-life; 2.0, 6.0, 20.0 mg/m^2^ b.s.a. are equivalent to 0.1, 0.3 or 1.0 mg tTF-NGR/kg b.w./day.
